# Resource Allocation for Epidemic Control in Metapopulations

**DOI:** 10.1371/journal.pone.0024577

**Published:** 2011-09-13

**Authors:** Martial L. Ndeffo Mbah, Christopher A. Gilligan

**Affiliations:** 1 Yale School of Public Health, Yale University, New Haven, Connecticut, United States of America; 2 Department of Plant Sciences, University of Cambridge, Cambridge, United Kingdom; Massey University, New Zealand

## Abstract

Deployment of limited resources is an issue of major importance for decision-making in crisis events. This is especially true for large-scale outbreaks of infectious diseases. Little is known when it comes to identifying the most efficient way of deploying scarce resources for control when disease outbreaks occur in different but interconnected regions. The policy maker is frequently faced with the challenge of optimizing efficiency (e.g. minimizing the burden of infection) while accounting for social equity (e.g. equal opportunity for infected individuals to access treatment). For a large range of diseases described by a simple SIRS model, we consider strategies that should be used to minimize the discounted number of infected individuals during the course of an epidemic. We show that when faced with the dilemma of choosing between socially equitable and purely efficient strategies, the choice of the control strategy should be informed by key measurable epidemiological factors such as the basic reproductive number and the efficiency of the treatment measure. Our model provides new insights for policy makers in the optimal deployment of limited resources for control in the event of epidemic outbreaks at the landscape scale.

## Introduction

The management of diseases involves the expenditure of limited resources, which more often than not are outstripped by the demand for controlling all infected individuals [1]–[3]. This is often the case when disease occurs simultaneously in different but inter-connected regions [2], [4], [5]. Treatment of infection in one region such as a state, city, or hospital may affect the potential for spread to another region when there is movement of individuals between the regions. Seeking to control disease outbreaks in more than one region, poses a dilemma for epidemiologists and health administrators of how best to deploy limited resources, such as drugs or trained personnel, amongst the different regions [6]–[11]. One common objective is to minimise the numbers of infected individuals and hence to minimize the burden of infection during the course of an epidemic [4], [12]. For epidemics of the SIS (Susceptible-Infected-Susceptible) form, in which individuals can be re-infected, Rowthorn et al. [10] showed that rather than targeting the region with most infecteds, as might have been intuitively expected, it is instead optimal to give preference to treating the region with the *lower* levels of infecteds: the remaining regions are treated as residual claimants, receiving treatment only when there is resource left over. The epidemiological intuition underpinning the optimal strategy is understood by noting that since there are only two types of host (susceptible or infected), preferential treatment in a region with low level of infection is equivalent to giving preference to the region with the highest level of susceptibles available for infection. Since, on average an infected individual infects more than one susceptible, removing infecteds where susceptibles are plentiful reduces the force of infection of the epidemic and so is likely to bring the epidemic under control. But what happens when there are more than two epidemiological classes? For many diseases, reinfection is often preceded by a period of temporary immunity, yielding a third class of ‘removed’ individuals in the population that complicates the identification of an optimal strategy for control. In this paper, we focus on this much broader class of epidemics described by an SIRS model.

We consider an SIRS-type epidemic in which infected individuals cease to be infectious and move into a temporary immune (R) class, after which they become susceptible once again. This is characteristic of many diseases, such as malaria [13], [14], tuberculosis [15] and syphilis [16], in which infecteds (I) recover naturally or after treatment. Infected individuals gain a temporal immunity to the pathogen, after which they rejoin the susceptible class (S) and can be reinfected. We assume that treatment is not used as a prophylactic so that only infected individuals receive treatment. Hence, the proportion of treated individuals is given as 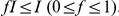


To address the problem of resource allocation for disease management in multiple regions, we use a combination of optimization methods from economic theory of disease control [17], [18] with a metapopulation model from epidemiological theory [19], [20]. This enables us to formalize the problem and to derive criteria for optimality so as to minimize the total number of infections over time. Not infrequently, strict criteria for optimization identify strategies that may be logistically impractical, for example by requiring a change in pattern of control at a switching time that may be difficult to monitor [17]. Strictly optimal strategies may also be challenged on grounds of social equity, whereby every infected individual does not have an equal chance of being treated [21], [22]. Accordingly we assess the tractability of optimal control strategies and consider also how adaptations may be made to balance, optimality, tractability and social equity. For the sake of simplicity, the analysis is initially carried out for two interconnected regions (e.g.cities, towns or states) and the robustness of the results to spatial structure are later tested for two other simple and realistic spatial configurations.

### Model

We consider two coupled sub-populations (regions) of susceptible individuals each with a fixed size 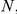 in which an epidemic is described by a simple SIRS compartmental model:
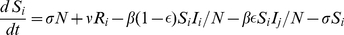
(1)

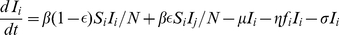
(2)

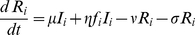
(3)with 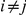 and 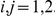 Each sub-population is composed of susceptible (S), infectious (I) and recovered (R) individuals, and are scaled here as proportions. The transmission rate for each sub-population is given by 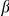. The coupling strength between sub-populations is given by 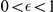. The infectious period is given by 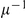; 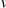 is the rate of loss of immunity, and 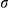 is rate of birth/death. 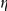 is a measure of the incremental increase in the recovery rate of treated individuals, and 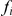 is the proportion of infected individuals in sub-population 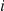 that receive treatment. When all infected individuals receive treatment 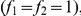 the basic reproductive number, which is a widely-used epidemiological measure of the intrinsic potential for multiplication of an epidemic, is given by 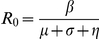. Without treatment 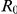 is equal to 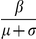.

### Optimal control

We suppose that expenditure on control is subject to a budget constraint 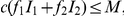 where 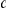 is the cost of treatment per individual. This simple fixed budget constraint is used as a surrogate that encompasses limitations in the amount of drug available and for mobilisation and delivery of resources at the point of infection (limitations in transport or trained personnel). These limit the instantaneous availability of drug. If there are sufficient resources, all infected individuals will be treated. Otherwise, resources are allocated so as to minimize the discounted number of infected individuals in both sub-populations over time. Hence, we choose 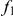 and 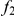 so as to minimize the following integral
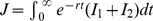
(4)


The discount rate (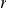) is included to allow for long-term changes, thus giving greater emphasis to control in the short rather than the long term [17]. The optimization approach we adopt is based upon the Hamiltonian method [23], which is a device for minimizing the objective function subject to the economic constraints and the epidemiological dynamics of the model.

We assume that if it were possible to treat all infected individuals, disease eradication would be achieved in the long term (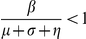). Using Filippov's theorem [24], it is possible to show that the optimal control problem does have a solution. To solve the problem of optimal deployment of limited resources (i.e., when there are insufficient resources to treat all individuals that may become infected), we use the Pontryagin maximum principle [23](PMP), a mathematical tool widely used to solve optimal control problems for dynamical systems. This method takes into account the influence of current infection on the future evolution of disease as given by the propagation equations (1)–(3). The influence is embodied in the co-state variables that appear in a mathematical expression known as the Hamiltonian (see Materials and Methods). PMP enables us to derive necessary conditions for optimality from which it is possible to build up a set of candidate strategies for optimality from which ultimately it is possible using extensive numerical simulation to identify an optimal solution.

## Results

### Efficiency maximization

The Pontryagin maximum principle (PMP) was used to derive necessary conditions for optimal resource allocation, when there are insufficient resources to treat all infected individuals. Using these necessary conditions together with exploratory numerical analysis, we identify the following as candidate strategies for optimality (see Materials and Methods):

- preferential treatment of the more infected sub-population - to equalize disease burden within the regions as fast as possible and thereafter to treat each region equally;
- preferential treatment of the less infected sub-population - initially ‘sacrificing’ the sub-population with the higher level of infecteds
- preferential treatment of the more susceptible sub-population - initially ‘sacrificing’ the sub-population with the lower level of susceptibles
- a strategy involving at least one switch between preferential treatment of the more infected to either the less infected or the more susceptible sub-population.

**Figure 1:**
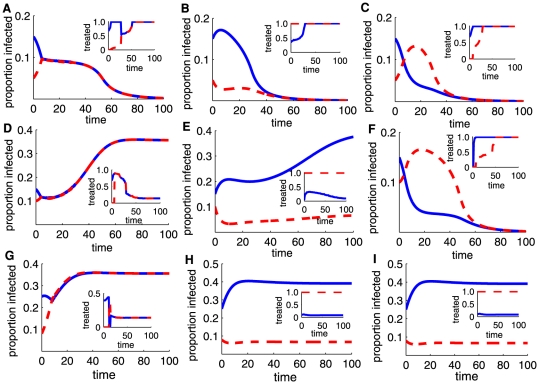
Comparison of disease progress curves for a strategy that gives preferential treatment to the more infected sub-population (A,D,G), preferential treatment to the less infected sub-population (B,E,H) and the most efficient strategy (C,F,I). Disease progress is shown for different control outcomes. (A–C) Progress of disease in two interconnected regions 1 (solid lines) and 2 (dashed lines), with treatment dynamics in insets, showing little differences between control strategies. (D–F) Preferential treatment to the more infected sub-population and preferential treatment to the less infected sub-population diverge markedly from the most efficient strategy. (G–I) Disease continues to increase but markedly less steeply in the region with the lower infestation (region 2), for preferential treatment to the less infected sub-population and most efficient strategy. Disease progress curves are given for three different values of the initial number of infected: (A,B,C), (D,E,F), and (G,H,I). Default parameters are 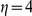 (efficiency of control), 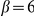 (within-region transmission rate), 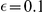 (coupling strength), 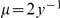 (recovery rate), 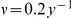 (rate of loss of immunity), 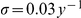 (rate of birth/death), 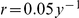 (discount rate), and 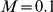 (fixed expenditure limit).

**Figure 2:**
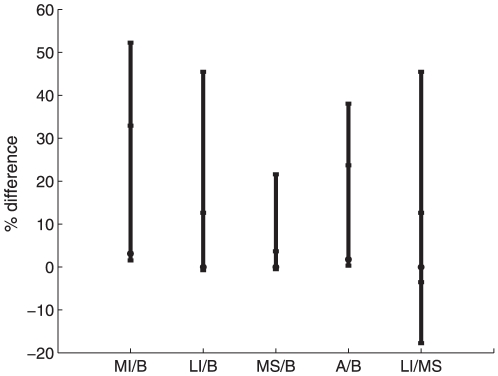
Difference between the outcome of the different policies for the whole range of initial conditions. For a given initial condition, the difference between two strategies (e.g ‘preferential treatment to the more infected sub-population’ and ‘preferential treatment to the less infected sub-population’) is computed by 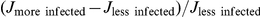 in which 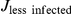 and 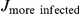 are the values of the discounted burden of infection for the ‘preferential treatment to the more infected sub-population’ and ‘preferential treatment to the less infected sub-population’ strategy, respectively. MI, MS, LI, A, and B denote respectively the ‘preferential treatment to the more infected sub-population’, ‘preferential treatment to the more susceptible sub-population’, ‘preferential treatment to the less infected sub-population’, alternative and single switch policy from ‘preferential treatment to the more infected sub-population’ to ‘preferential treatment to the less infected sub-population’. The average difference value is represented by the dot, whereas the top and bottom bars represent respectively the maximum and minimum values. As for the middle bars, they represent respectively the ninety-ninth and first percentiles. The average and percentiles were obtained for 705078 initial conditions (See Materials and Methods).

Although it is not possible to prove analytically that a given path is optimal, after extensive numerical simulation, we identify the single switch strategy from giving preference to the more infected sub-population to giving preference to the less infected sub-population as the best allocation strategy that minimizes the discounted total numbers of infected individuals in both sub-populations (Figs. 1 & 2). However, attempts to implement the switching strategy are prone to the risk of missing the optimal switching time. This risk is enhanced by the fact that the optimal switching time depends upon the values of epidemiological parameters and the initial levels of infection that are unlikely to be accurately known in advance.

To conclude our analysis on efficiency maximization, we investigate the effect of the rate of loss of immunity 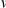 on the best allocation policy, by considering respectively the cases 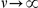 and 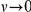 (see Table 1). Using numerical simulation, we compare the candidate strategies for optimality (see Materials and Methods) and show that for very large values of 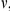 the best allocation strategy is always to give ‘preference to the more susceptible sub-population’. This observation agrees with the results of Rowthorn et al. [10] who show this policy to be the best strategy for the control of an SIS type epidemic. Whereas for very small values of 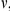 a double switch of preference between the more and the less infected region was shown to outperform the other allocation strategies. It is difficult to prove the existence of an upper bound to the number of switches. However, the more switches there are, the harder the implementation of the allocation strategy would be.

**Table 1:** Effect of the rate of loss of immunity (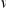) on the best allocation strategy.

Epidemic model	SIRS	SIRS 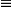 SIS; 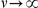	SIRS 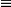 SIR; 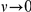
Best strategy	‘single switch^1^’	‘no switch^2^’	‘double switch^3^’

1 single switch of preference from the more infected sub-population to the less infected sub-population.

2 preference to the less infected sub-population.

3 double switch of preference between the less infected and the more infected sub-population.

**Figure 3:**
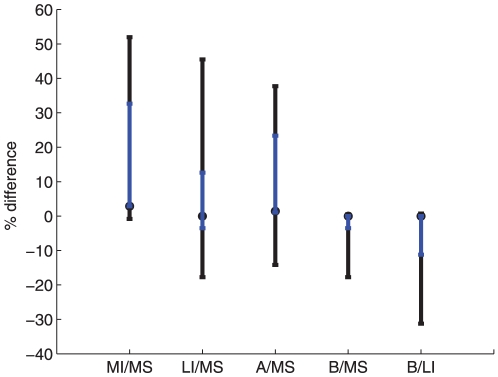
Difference between the outcome of the different policies for the whole range of initial conditions. Notations follow from Fig. 2. The figure shows the difference in efficiency between ‘preference to the more infected sub-population’ and ‘preference to the less infected sub-population’ policies. The average value is represented by the dot, whereas the top and bottom black bars represent respectively the maximum and minimum values. The middle blue bars represent respectively the ninety-ninth and first percentiles.

**Figure 4:**
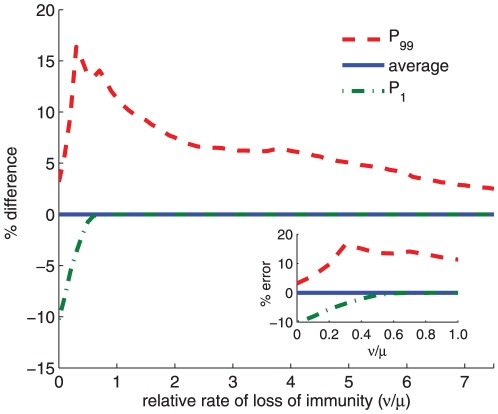
Difference between the outcome of ‘preferential treatment to the less infected sub-population’ and that of ‘preferential treatment to the more susceptible sub-population’. The figure shows the effect of the change of the rate of loss of immunity on the difference between the outcome of the two strategies. The inset shows the effect for values of the rate of loss of immunity 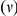 less or equal to the natural recovering rate 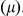 Difference is computed as with Fig. 2. The solid line represents the average value, whereas the dashed and dash dotted lines represent respectively the ninety-ninth and the first percentiles. The average and percentiles were obtained for 705078 initial conditions (See Materials and Methods).

The single switch strategy, though the best policy, is not easily implementable. Numerical simulation shows that the second best policy in terms of simplicity and efficiency maximization is either to give preference to the more susceptible sub-population or preference to the less infected sub-population depending on the initial state of the system (Fig. 3). We compare the performance of these policies for different values of the rate of loss of immunity 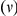 (Fig. 4). For 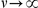 the two inequitable policies are identical. As the value of 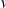 decreases, the difference between the policies increases, with the preferential treatment of the more susceptible sub-population outperforming the preferential treatment of the less infected sub-population. However, when the rate of loss of immunity becomes small 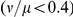 the relative performance of the two policies becomes highly dependent on the initial state of the system (Fig. 4).

### Efficiency and social equity

Since the optimal strategy is very difficult to implement, two robust alternative strategies would be either to give preference to the more susceptible sub-population or to give preference to the less infected sub-population. However, these strategies are likely to be regarded as highly socially inequitable from the perspective of the chance that any infected individual receives treatment. For the initial state of the system satisfying: 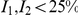 and 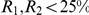 (which may be regarded as early implementation of control), we consider a widely-advocated, socially equitable strategy comprising

- a pro-rata policy designed to give equal opportunity for any infected individual to receive treatment [22], [25].

**Figure 5:**
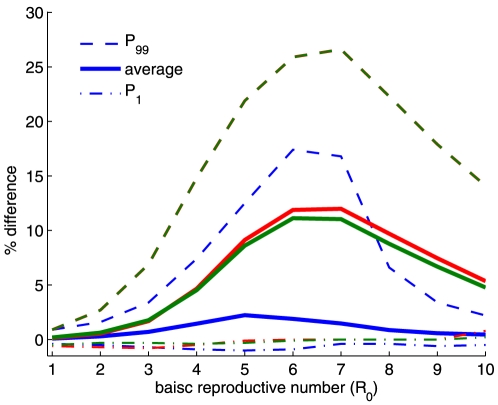
Difference between the outcome of selected strategies for different values of *R*_0_. The blue lines represent the difference between the outcome of ‘preferential treatment to the more infected sub-population’ and that of the pro-rata policy. The green lines represent the difference between the outcome of the pro-rata policy and that of ‘preferential treatment to the more susceptible region’, and the red lines represent the difference between the outcome of the pro-rata policy and that of ‘preferential treatment to the less infected region’. Averages are obtained for 10,000 initial states of the system, generated randomly.

We compare the performance of this strategy with the three tractable strategies considered above (i.e. not involving switching). We do this for different values of the basic reproductive number 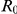 (Fig. 5): 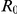 is a widely-used epidemiological measure of the intrinsic potential for multiplication of an epidemic.

**Figure 6:**
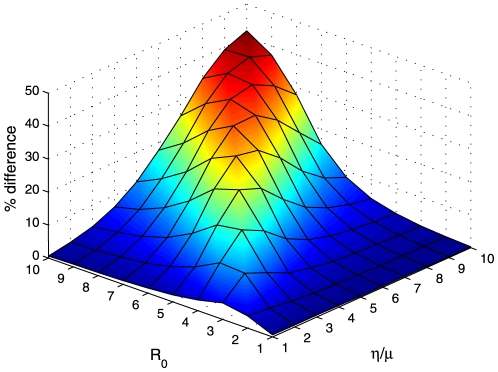
Difference between the outcome of ‘preferential treatment to the less infected sub-population’ and that of that pro-rata policy for symmetrical global connection between regions. The effect of the efficiency of the treatment measure on the average difference of outcome between ‘preferential treatment to the less infected sub-population’ and pro-rata policy with respect to the basic reproductive ratio 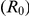 is shown. Averages are obtained for 10,000 initial states, generated randomly, of a system of 10 regions. Default parameter values as given in Fig. 1, except 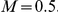

Given a threshold value, of the difference between the outcome of a given control strategy and that of the pro-rata strategy, (d%) above which the use of inequitable policies may be justifiable, Fig. 5 shows that there exists a threshold value 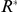 such that for 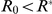, the pro-rata policy performs almost as well as the other policies (e.g. for d = 10% 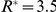). In this case, the pro-rata policy is a good compromise in terms of equity, efficiency and simplicity. For 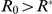, it would be better to opt for an inequitable policy (e.g. preferential treatment to the more susceptible sub-population). For high values of 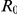, the decreasing difference of value between the policies is due to the inefficiency of the control measure (drug efficiency) in bringing the epidemic under control (cf. Fig. 5 and 6). We also compare the policies for different value of the coupling strength between the two sub-populations 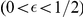. We observed that when the coupling strength decreases, the difference between the outcome of the control policies increases with the pro-rata strategy becoming the more less efficient than the optimal strategy (the single switch strategy from giving preference to the more infected sub-population to giving preference to the less infected sub-population). On the other hand, the difference between the outcome of the control policies declines as the coupling strength gets larger. Thus, as the transmission between the sub-population increases the outcome becomes less sensitive to the choice of policy [10] (result not shown here).

**Figure 7:**
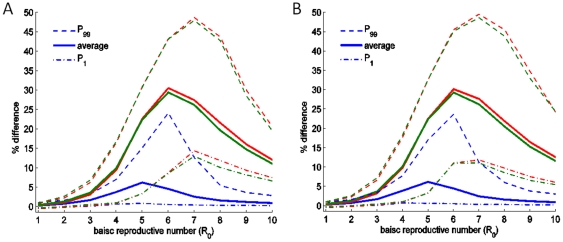
Difference between the outcome of selected policies for different values of *R*_0_ for multiple sub-populations with different coupling between sub-populations. For (A), regions are inter-connected with symmetrical global coupling. For (B), regions are inter-connected only with nearest neighbours. The blue lines represent the difference between the pro-rata policy and the ‘preferential treatment to the more infected sub-population’ policy. The green lines represent the difference between the ‘preferential treatment to the more susceptible sub-population’ policy and the pro-rata policy, and the red lines represent the difference between the ‘preferential treatment to the less infected sub-population’ policy and the pro-rata policy. Averages are obtained for 10,000 initial states, generated randomly, of two systems of 10 regions. Default parameter values as given in Fig. 1, except 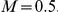

**Figure 8:**
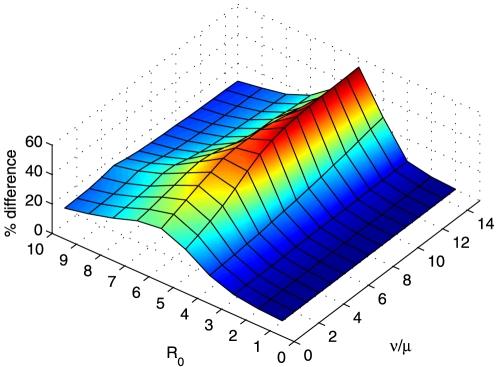
Difference between the outcome of ‘preferential treatment to the less infected sub-population’ and that of the pro-rata policy for symmetrical global connection between regions. The effect of the change of the rate of loss of immunity on the average difference between ‘preferential treatment to the less infected sub-population’ and pro-rata policy with respect to the basic reproductive ratio 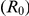 is shown. Averages are obtained for 10,000 initial states, generated randomly, of a system of 10 regions. Default parameter values as given in Fig. 1, except 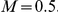

To investigate the robustness of the result to spatial structure, we consider two further spatial configurations: 10 identical regions with symmetrical global coupling, and 10 identical regions arranged in a circle with each population interacting only with its two nearest neighbours. For small values of 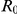, the relative outcome of the control policies is independent of the spatial structure of the system (Fig. 7). But for high values of 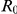, the variability in the outcome of the control policies with respect to the initial state of the system increases with the sparsity of the coupling matrix (Fig. 7). Simulation shows that the threshold value of 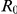 increases with the efficiency of the treatment measure (Fig. 6), and decreases for increasing values of the rate of loss of immunity (Fig. 8). Given that the choice of the discount rate affects the relative valuation of the current and future disease, one would expect a correlation between the choice of the discount rate and the value of the percentage error above which social inequity is justifiable.

When pro-rata is not a good candidate strategy in terms of efficiency 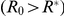, an alternative strategy for balancing efficiency and social equity may be the use of proportional allocation where a fraction of the resources is allocated pro-rata, while the remaining is allocated so as to maximize efficiency [22]. However, determining what fraction of resource is to be allocated for equity concerns, while retaining a good level of overall efficiency, requires further debate and greater interrogation of epidemiological models with insight from social sciences [22], [26].

## Discussion

We have addressed the problem of allocation of limited resources for the control of an SIRS-type epidemic in different but interconnected regions. Using a combination of optimization methods from economic theory with a metapopulation model from epidemiological theory for disease management, we have formalized the problem of resource allocation and derived criteria for optimality so as to minimize the discounted number of infected individuals in both sub-populations over time, during the course of the epidemic. Using extensive numerical simulations, we have shown that the best strategy in terms of efficiency maximization is a switching strategy, whereby resources are initially preferentially allocated to the more infected sub-population then to the less infected sub-population. However, this strategy is seldom tractable, due to the fact that the switching time depends upon the value of epidemiological parameters and the initial state of the system, which are unlikely to be accurately known [17], [27].

Given that a practical strategy for disease control must account for various factors such as efficiency maximization and social equity amongst others, we have extended previous studies on dynamic resource allocation by investigating how to account for optimality (minimizing the burden of infection), social equity (equal opportunity for infected individuals to access treatment), and simplicity (ease of implementation) in identifying strategies for disease control. We have shown that when faced with the dilemma of choosing between a socially equitable strategy for resource allocation (e.g. a pro-rata allocation strategy) and a purely efficient but inequitable strategy (e.g. by giving preference to the more susceptible sub-population or preference to the less infected sub-population), the decision should be informed by the value of key epidemiological and economic parameters. In particular, we have shown that given a certain percentage of difference between the outcomes of different strategies (i.e. relative discounted number of infections that are not averted under the pro-rata policy) above which the use of an inequitable policy may be justifiable, there exists a threshold value of the basic reproductive number (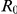) below which it is better to adopt a purely socially equitable strategy (pro-rata policy). This threshold value was shown to increases with the efficiency of the treatment measure, and to decrease with the average duration of the period of temporal immunity. The social context of our analysis implies that equal weighting is given to the health of each individual i.e. for the collective good of the entire population.

Interest in the optimal allocation of resources for epidemic control in structured populations has recently been renewed due to the threat of pandemic influenza [11], [28]–[30]. These studies primarily focus on the optimal deployment of mass vaccination to prevent or mitigate the spread of an outbreak of influenza within a population. Among other things, they show that when vaccine supplies are limited and the public health objective is to minimize infections, it is optimal to target vaccination toward the more epidemiologically important sub-populations (those that suffer the greatest per capita burden of infection) [11], [28]–[30]. The other sub-populations would thus be indirectly protected through herd immunity [11], [28]. These results agree with our analysis which shows that a good control strategy in terms of simplicity and efficiency maximization would be to give preference to the more susceptible sub-population. This sub-population may be regarded as the more epidemiologically important as it is potentially the main contributor to future infections.

Several areas of investigation suggest themselves for future work. Foremost amongst these are allowance for heterogeneity in the size of sub-populations, and the rates of transmission of infection, both of which are recognized to be important factors in metapopulation theory. Further work will also investigate the robustness of the results for different measures for efficiency of control and to uncertainty about the likely values of epidemiological parameters, given that optimal strategies are often very sensitive to the epidemiological parameters [27], [31]–[33], which may not be accurately known before control is implemented.

## Materials and Methods

The objective is to minimize the discounted burden of infection during the course of the epidemic
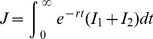
(5)subject to the propagation equations (1)–(3)and the following epidemiological and economic constraints:
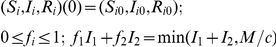



Each sub-population, of a fixed size 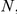 is composed of susceptible (S), infectious (I) and recovered (R) individuals, and are scaled here as proportions.

Let 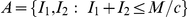 be the region where there are sufficient resources to treat all infected individuals. Since 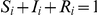, the equations on 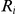 can be ignored.

When there are more infecteds that can be treated, 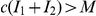 and hence 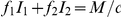. The relevant Hamiltonian in this case is
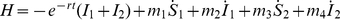
(6)where 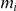 are the co-state variables. Since 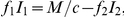 the Hamiltonian can be written as
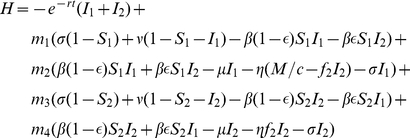
(7)



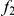
 (and hence 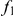) has to be chosen so as to maximize the Hamiltonian [23]. This yields the following result:
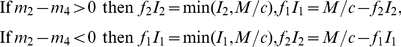
(8)


And it must be the case that
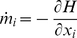
(9)where 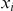 is the corresponding state variable to 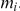


We assume that 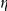 satisfies the following condition:
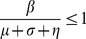
(10)


That 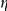 satisfies equation 10 implies that if there are always enough resources to treat all infected individuals, disease will eventually be eradicated in the population. This is justified by the fact that this criterion (equation 10) is equivalent to the basic reproductive ratio 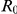 being less than or equal to 1 [34], which here is a necessary and sufficient criterion to prevent invasion of an epidemic. Upon equation 10 any admissible path (disease dynamic curves obtained for a given value of the control functions 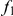 and 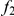) will either never enter region 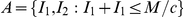, or enter and never leave (see [10] and [27] for details). Therefore, besides the general transversality conditions 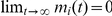, there are alternative transversality conditions whenever a path enters region 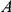 [23]. We define a function 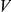 as follows
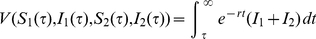
(11)where the integral is evaluated along the path defined by the propagation equations (1)–(3) when 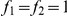, with 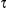 being the time at which the path enter region 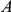. The alternative transversality conditions for a path that enters region 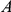 is given by
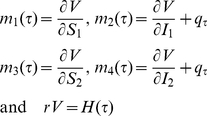
(12)where 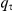 is a multiplier, and 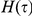 is the Hamiltonian evaluated at time 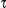.

Given an initial state of the system 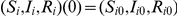, the existence of an admissible path which enters region 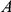 depends upon the value of the expenditure limit 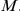 When such a path exists, the optimal control problem is equivalent to an optimal timing problem, where the objective is to find the shortest path to reach region 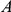. For such a value of 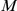, a simple application of Filippov's theorem [24] shows that a solution to the optimal control problem exists. This is done using Theorem 10.1 from [24], and the compactness of the set of points 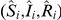 at which admissible paths, starting at 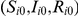, enter region 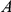.

### The singular solution

We suppose that there exists an allowable path that satisfies the above maximal conditions on the Hamiltonian, and for which there exists an open interval where we have 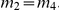 By differentiating 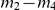 over that open interval, we obtain
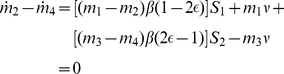
(13)


From an economical view point, the co-state variables can be interpreted as shadow prices. Thus 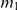 and 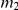 indicate respectively the marginal benefit to society of increasing by one unit the proportion of susceptible (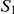) and infectious (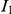) individuals of region 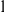 [35], [36]. Because infection is harmful, and increasing the proportion of infectious individuals will result in decreasing the proportion of susceptibles, the shadow price 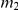 is negative. Then, 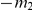 represents the proportion that society is willing to invest for control that will result in reducing the stock of infectious individuals in region 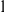 by one unit. Moreover 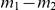 is positive. The same results hold for 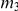 and 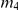. Since 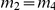 on an open interval, it follows that 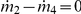 on such an interval. Equation 13 is then equivalent to
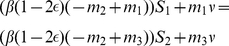
(14)


From (14), it follows that 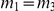 if and only if 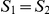. If 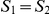 on an open interval, it follows from the previous sentence that we would have 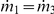 on the same open interval. Simple algebra shows that with 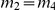 and 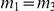 on an open interval, 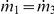 implies that 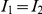 on the same interval. Therefore, if there exists an open interval on which 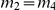 and 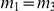, then 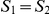 and 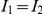 on the same interval. The control strategy on such an interval would be given by 
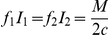
.

Since 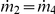 on an open interval, it follows from equation 9:
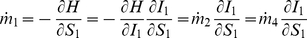


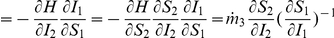



From the symmetry of the system, we have 
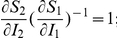
 then 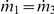 on the open interval. We conclude from the transversality conditions that 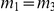. It follows that the singular solution is given by:
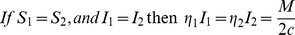
(15)which satisfies that following equations:
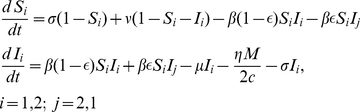
(16)


The singular solution is achieved by preferential treatment of infecteds in the region with the higher prevalence of infecteds (see Eq. 17). The policy is called the MRAP since it involves the Most Rapid Approach Path to the singular solution, in which infection is equalized in both sub-populations.

When 
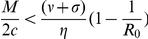
 (where 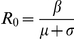) equation (16) has two equilibrium points given by
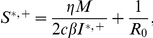
and
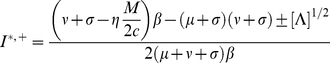
with 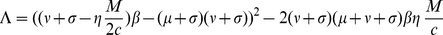. We have 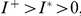 where 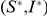 is unstable (saddle point), and 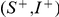 is stable. Another stable equilibrium point (disease free equilibrium) is reached if the path enters region A.

**Figure 9:**
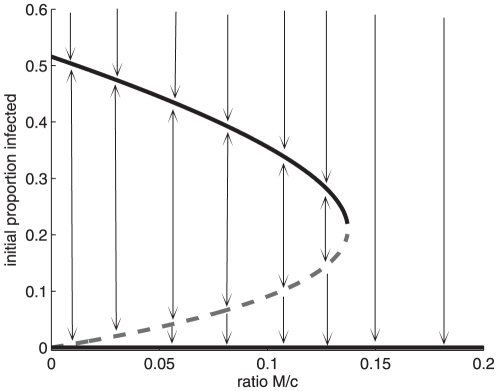
Bifurcation diagram for the singular solution (Eq. 16). The dashed line represents unstable steady states and the solid lines represent stable steady states. Using the most rapid approach path to the singular solution (MRAP), the initial proportion of infected represents the level of infection when the singular solution is first reached. Parameter values are given by Fig. 1.

When 
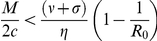
, the singular solution may exhibit a saddle-node bifurcation along the bifurcation parameter 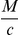 (see Fig. 9). In other words, when the average proportion of individuals treated individuals in each sub-population 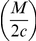 is lower than the epidemiological factor 
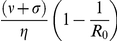
, the singular solution fails to eradicate the disease, as the infection path converges towards 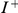, if control strategy (MRAP) is first implemented when the proportion of infected in both sub-population is above the unstable steady state (dashed line in Fig. 9).

### Candidates for optimality

From the above results, it follows that the optimal control strategy depends on the effect of a marginal change in the value of 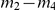. However, this change can only be determine numerically. Using the shadow pricing analogy together with exploratory numerical analysis, we derive some scenarios of practical understanding that can be understood in terms of the co-state variables 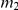 and 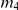.

From the interpretation of the co-state variables as shadow prices, equation (8) can be interpreted as follows: if increasing the amount of infected individuals in sub-population 1 (sub-population 2) by one unit, would generate more infection in the whole population than an increase of the same amount in sub-population 2 (sub-population 1), then preference in treatment must be given to sub-population 1 (sub-population 2). From equation (15) and equation (8), it follows that an optimal solution is either a switching strategy of preference between sub-population 1 and sub-population 2, or the MRAP (the most rapid approach path to singular solution). The MRAP solution, which is equivalent to ‘preferential treatment of the more infected sub-population’ is given by the following equation:
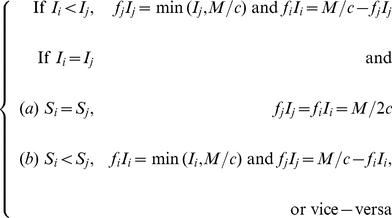
(17)


As for the switching strategies between sub-population 1 and 2, they can be constructed in an infinite number of ways. Here we consider two plausible candidate solutions for optimality (based upon exploratory numerical analysis): ‘preferential treatment of the more susceptible sub-population’, ‘preferential treatment of the less infected sub-population’. These strategies are respectively defined by the following equations:
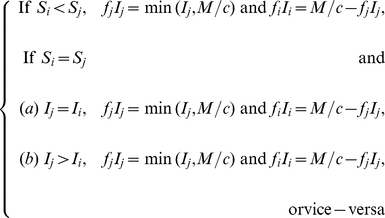
(18)and
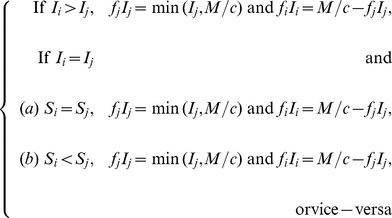
(19)


The strategies giving preference to the more susceptible sub-population, preference to the less infected sub-population as well as the single and double switching strategies between one of the above strategies and the MRAP strategy are all candidates for optimality. Moreover, we consider an ‘alternative’ strategy which consists in the first instance in equalizing the level of infection in both sub-populations as fast as possible. This is done by implementing the strategy giving preference to the more infected sub-population strategy. When equality of the levels of infection is first reached, preference is then given to the more susceptible sub-population. We compare the above strategies. For any value of the initial condition, simulation shows that the smallest value of the objective function (Eq. 5) is obtained with the single switch strategy from giving preference to the more infected sub-population to giving preference to the less infected sub-population. Implementing the single switch strategy is subject to the risk of missing the optimal switching time. We were also able to show that the switching strategy satisfies the Hamiltonian and transversality conditions. We were not able to rule out the possibility that there are other paths, such multiple switching strategies, which outperform the above strategy.

Simulation shows that the optimal switching strategy varies with the rate of loss of immunity 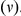 For 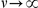 (SIRS equivalent to an SIS model), the best allocation strategy is always to give preference to the less infected sub-population (here giving preference to the less infected sub-population is equivalent to giving preference to the more susceptible sub-population). This observation agrees with Rowthorn et al. [10]. For 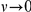 (SIRS equivalent to an SIR model), a double switch of preference between the less and the more infected sub-population outperforms the other allocation strategies.

### Details of the numerical explorations

Numerical simulation was done using a fourth order Runge-Kutta scheme with 0.01 time intervals. Experiments were done for different values of the period of integration and time intervals. The accuracy of our method was established up to three decimal places. The state variables were scaled with respect to the fixed sub-population size 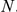


To compare different control strategies, simulations were done using a large set of initial conditions (the state of the epidemic in each sub-population before resources are first allocated). For every single initial condition, we compared the value of the objective function for each of the control strategies described above. To build the set of initial condition, we proceeded as follows: for each sub-population, we spanned the surface 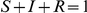 using an increment step of 0.02, excluding extreme cases such as 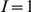 and 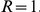 By crossing the initial conditions for the two sub-populations, we obtain a set of 705078 initial conditions for the whole system. The optimality of the single switch strategy was shown to hold for all initial conditions.

Comparing the proposed candidates for optimality is not enough to establish the optimality of a given solution. We used the same method as Rowthorn et al. [10]. We consider the paths that eventually reach set 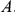 Any such path crosses the frontier of 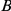 at a unique point 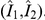 At this point, the transversality conditions determine a unique set of shadow prices 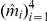. Taking 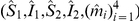 as initial conditions, we can reverse the systems of equations (1) and (9), thus tracking a path backward out of set 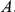 Reversing a second time converts this path into a unique forward path that meets the set 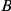 at 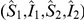 and also satisfies the Hamiltonian and transversality conditions. Using various points on the frontier of 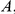 and suitable values of 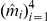, we were not able to find another solution, satisfying the Hamiltonian and transversality conditions, that outperforms the switching strategies (also known as ‘bang-bang’ solutions [23]).

## References

[1] Lipsitch M, Bergstrom CT, Levin B (2000). The epidemiology of antibiotic resistance in hospitals: Paradoxes and prescriptions.. Proc Natl Acad Sci USA.

[2] Kiszewski A, Johns B, Schapira A, Delacollette C, Crowell V (2007). Estimated global resources needed to attain international malaria control goals.. Bull World Health Organ.

[3] Monto A (2006). Vaccines and antiviral drugs in pandemic preparedness.. J Infect Dis.

[4] Dye C, Gay N (2003). Epidemiology: modeling the SARS epidemic.. Science.

[5] Sani A, Kroesea D (2008). Controlling the number of HIV infectives in a mobile population.. Math Biosci.

[6] May R, Anderson R (1984). Spatial heterogeneity and design of immunization programs.. Math Biosci.

[7] Hethcote H, van Ark J (1987). Epidemiological models for heterogeneous populations: proportionate mixing, parameter estimation, and immunization programs.. Math Biosci.

[8] Zaric G, Brandeau M (2002). Dynamic resource allocation for epidemic control in multiple populations.. IMA J Math Appl Med Biol.

[9] Brandeau M, Zaric G, Ricther A (2003). Resource allocation for control of infectious diseases in multiple independent populations: beyond cost-effectiveness analysis.. J Health Econ.

[10] Rowthorn R, Laxminaryan R, Gilligan C (2009). Optimal control of epidemics in metapopulations.. JRSoc Interface.

[11] Keeling M, White P (2010). Targeting vaccination against novel infections: risk, age and spatial structure for pandemic influenza in great britain.. http://dx.doi.org/10.1098.

[12] Dushoff J, Plotkin J, Viboud C, Simonsen L, Miller M (2007). Vaccinating to protect a vulnerable subpopulation.. PLoS Med.

[13] Aron J (1988). Mathematical modeling of immunity to malaria.. Mathematical Biosciences.

[14] Filipe J, Riley E, Drakeley C, Sutherland C, Ghani A (2007). Determination of the processes driving the acquisition of immunity to malaria using a mathematical transmission model.. PLoS Comput Biol.

[15] Castillo-Chavez C, Feng Z (1997). To treat or not to treat: the case of tuberculosis.. J Math Biol.

[16] Grassly N, Fraser C, Garnett G (2005). Host immunity and synchronized epidemics of syphilis across the united states.. Nature.

[17] Forster G, Gilligan C (2007). Optimizing the control of disease infestations at the landscape scale.. Proc Natl Acad Sci USA.

[18] Goldman S, Lightwood J (2002). Cost optimization in the SIS model of infectious disease with treatment.. Top Econ Anal Policy.

[19] Hanski I (1998). Metapopulation dynamics.. Nature.

[20] Keeling M, Grenfell TD (2000). Individual-based perspectives on R0.. J Theor Biol.

[21] Strosberg M (2006). Allocating scarce resources in a pandemic: Ethical and public policy dimensions.. Virtual Mentor (Ethics J Am Med Ass).

[22] Kaplan E, Merson M (2002). Allocating hiv-prevention resources: balancing efficiency and equity.. Am J Pub Health.

[23] Seierstad A, Sydsaeter K (1986). Optimal control theory with economic applications..

[24] Agrachev A, Sachkov Y (2004). Control theory from the geometric viewpoint..

[25] HHS (2007). hhs pandemic influenza plan. Technical report, United States Department of Health and Human Services.. http://www.hhs.gov/pandemic/plan/sup6.html.

[26] Wu J, Riley S, Leung G (2007). Spatial considerations for the allocation of pre-pandemic influenza vaccination in the united states.. Proc R Soc Lond B.

[27] Ndeffo-Mbah M, Gilligan C (2010). Optimization of control strategies for epidemics in heterogeneous populations with symmetric and asymmetric transmission.. J Theor Biol.

[28] Medlock J, Galvani A (2009). Optimizing influenza vaccine distribution.. Science.

[29] Wallinga J, van Bovan M, Lipsitch M (2010). Optimizing infectious disease interventions during an emerging epidemic.. PNAS.

[30] Goldstein E, Apolloni A, Lewis B, Miller J, Macauley M (2010). Distribution of vaccine/antivirals and the ‘least spread line’ in a stratified population.. J R Soc Interface.

[31] Tanner M, Sattenspiel L, Ntaimo L (2008). Finding optimal vaccination strategies under parameter uncertainty using stochastic programming.. Math Biosci.

[32] Merl D, Johnson R, Gramacy B, Mangel M (2009). A statistical framework for the adaptive management of epidemiological interventions.. PLoS ONE.

[33] Ndeffo-Mbah M, Forster G, Wesseler J, Gilligan C (2010). Economically optimal timing of crop disease control in the presence of uncertainty: an options approach.. JRSoc Interface.

[34] Heffernan J, Simth R, Wahl L (2005). Perspectives on the basic reproductive ratio.. J R Soc Interface.

[35] Behncke H (2000). Optimal control of deterministic epidemics.. Optim Contr Appl Meth.

[36] Dorfman R (1969). An economic interpretation of optimal control theory.. Amer Econ Rev.

